# Scalable and versatile container-based pipelines for de novo genome assembly and bacterial annotation.

**DOI:** 10.12688/f1000research.139488.1

**Published:** 2023-09-25

**Authors:** Felipe Marques de Almeida, Tatiana Amabile de Campos, Georgios Joannis Pappas Jr

**Affiliations:** 1 Programa de Pós-graduação em Biologia Molecular, Universidade de Brasilia, Brasília, FD, 70910-900, Brazil; 2 Departamento de Biologia Celular, Universidade de Brasília, Brasília, DF, 70910-900, Brazil; 3 Programa de Pós-graduação em Biologia Microbiana, Universidade de Brasília, Brasília, DF, 70910-900, Brazil

**Keywords:** bacterial genomics, pipelines, nextflow, antibiotic resistance, public health, virulence

## Abstract

**Background:** Advancements in DNA sequencing technology have transformed the field of bacterial genomics, allowing for faster and more cost effective chromosome level assemblies compared to a decade ago. However, transforming raw reads into a complete genome model is a significant computational challenge due to the varying quality and quantity of data obtained from different sequencing instruments, as well as intrinsic characteristics of the genome and desired analyses. To address this issue, we have developed a set of container-based pipelines using Nextflow, offering both common workflows for inexperienced users and high levels of customization for experienced ones. Their processing strategies are adaptable based on the sequencing data type, and their modularity enables the incorporation of new components to address the community’s evolving needs.

**Methods:** These pipelines consist of three parts: quality control, de novo genome assembly, and bacterial genome annotation. In particular, the genome annotation pipeline provides a comprehensive overview of the genome, including standard gene prediction and functional inference, as well as predictions relevant to clinical applications such as virulence and resistance gene annotation, secondary metabolite detection, prophage and plasmid prediction, and more.

**Results:** The annotation results are presented in reports, genome browsers, and a web-based application that enables users to explore and interact with the genome annotation results.

**Conclusions:** Overall, our user-friendly pipelines offer a seamless integration of computational tools to facilitate routine bacterial genomics research. The effectiveness of these is illustrated by examining the sequencing data of a clinical sample of Klebsiella pneumoniae.

## Introduction

As whole genome sequencing has been established as a routine procedure in research projects worldwide, the computational analysis of sequencing data takes center stage, and it is often the main operational barrier to which biologists stumble ( Xuan et al., 2013; Berger and Yu, 2022). Over the years, many open-source software tools were created to tackle different processing steps along intricate computational protocols geared toward different data processing scenarios. Notwithstanding, materializing the data analysis workflow is a non-trivial stage that many biologists face, given challenges ranging from the selection and installation among a vast assortment of computational tools to the logic of enactment of processing steps. Consequently, analyses can be performed in many ways by different groups raising issues of reproducibility and provenance ( Grüning et al., 2018; Djaffardjy et al., 2023). Also, individual teams face problems implementing the processing workflow, inventorying the requirements, and optimizing performance and scalability ( Wratten et al., 2021; Mölder et al., 2021).

Bacterial whole-genome sequencing has become mainstream in many microbiological settings, such as taxonomy, ecology, and clinical diagnostics. In concert with these applications, several tailored computational workflows, also known as pipelines, were created for bacterial genomics, each with a different underlying design and implementation approach ( Petit and Read, 2020). Despite their similarities, each pipeline is unique and may provide different outcomes, considering that they were developed with different components and parameters. Most of the pipelines are designed to work with limited sequencing data types while focusing on specific annotation tasks, such as functional annotation of open reading frames, antibiotic resistance genes, and variant calling using reference genomes ( Olawoye et al., 2020; Quijada et al., 2019; Ruiz-Perez et al., 2021; Sserwadda and Mboowa, 2021). Thus, there is still a need for more generic pipelines that give the user an extensive overview of their data while creating visually rich outputs whilst guaranteeing reproducibility.

Here we describe three pipelines built using the workflow composition system Nextflow ( Tommaso et al., 2017). These were specifically designed in a modular way to standardize and facilitate bacterial genomic analysis from the standpoint of non-bioinformaticians relieving the issues of installation, configuration, and execution. Altogether, the pipelines are usable in different analytical scenarios, capable of handling data from different sequencing platforms, ranging from small single-genome projects executed on a personal computer to larger multi-genome projects to be executed in cloud computing platforms. We also leverage the use of operating system virtualization, packing the pipelines to use software containers that provide all required supporting programs without the need to install the required operating system and pipeline components.

Together, they offer a seamless exposition of computational tools to provide an easy framework for analyzing and interrogating data in routine bacterial genomics. To illustrate the system’s functionality, we provide a full analytical illustration of the processing of a multi-drug-resistant *Klebsiella pneumoniae* strain.

## Methods

### Implementation

The pipelines have been implemented with Nextflow, a workflow composition and orchestration tool that allows the execution of tasks across multiple heterogeneous computing environments in a portable manner ( Tommaso et al., 2017). We have used many concepts discussed and established by the nf-core community and adhered to their development framework ( Ewels et al., 2020).

In the pipeline design process, several auxiliary scripts were developed to aid in processing and summarizing intermediate steps and graphical annotation report generation. Several programming languages were used, like Python, R, RMarkdown, and Bash shell scripts. However, the utilization of scripting languages in the pipelines comes with the drawback of increased complexity, as it necessitates the installation of language interpreters and support libraries. To make this as transparent as possible to the end user, all the scripts and core dependencies have been packed into Docker ^®^ container images to ensure a consistent distribution and uniform execution regardless of the endpoint hardware and operating system.

**Figure 1.**
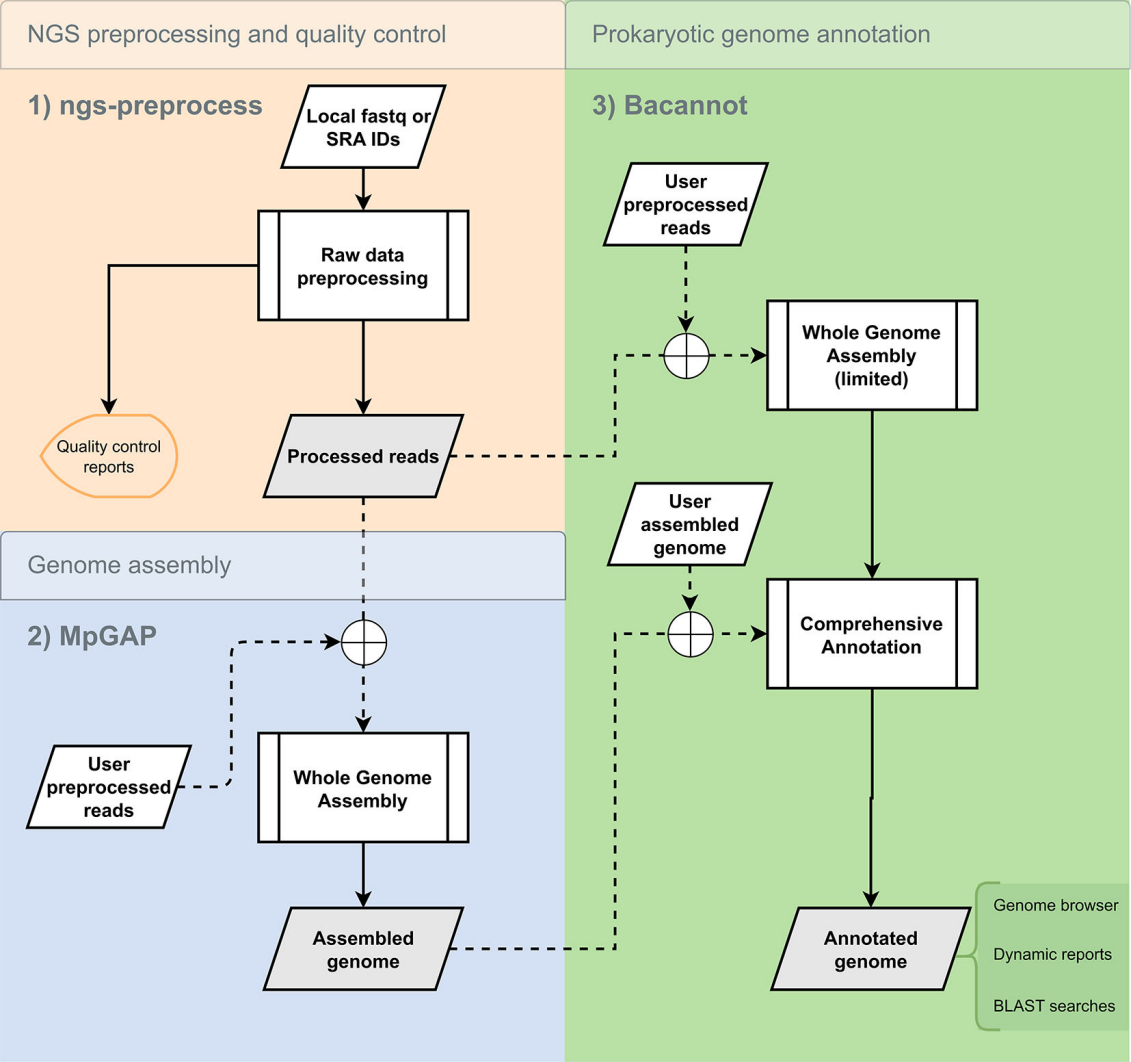
Flowchart of optional sequential execution of the developed pipelines for complete bacterial genomics analysis. The available whole genome assembly module in bacannot is considered limited compared to MpGAP, because it only contains two assembler options. Dashed arrows connected to the crossed circles represent the optional flow of data, highlighting that chaining the pipelines is not required. Gray boxes highlight the pipeline’s outputs.

We have adopted a modular development approach, resulting in three independent pipelines specializing in the critical steps of a general bacterial genomics pipeline ( Figure 1). The first pipeline focuses on data pre-processing and quality control, the second on genome assembly, and the third on genome annotation. Pipelines 1 and 2 can be used independently for any general-purpose sequencing project, while pipeline 3 is optimized for prokaryotic genomes. The pipelines are autonomous, meaning they can be used separately or in combination with other methods, giving users complete control and flexibility ( Figure 1). The pipelines provide a comprehensive end-to-end solution for bacterial genomics analyses when used sequentially. When invoked sequentially, the pipelines provide an end-to-end solution for bacterial genomics analyses. The architecture and implementation of each pipeline are detailed in the following sections.

### The preprocessing pipeline

**Figure 2.**
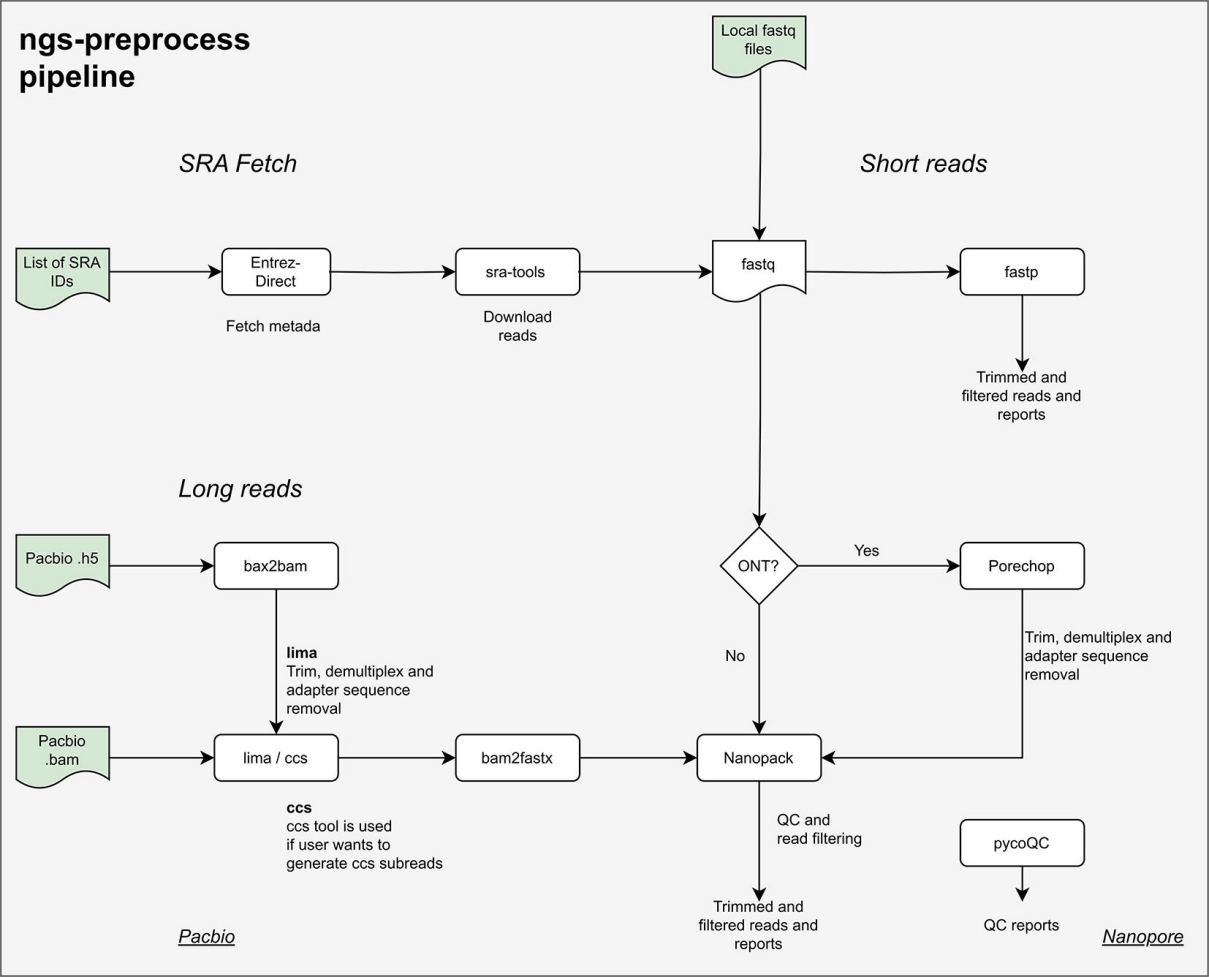
Flowchart of all steps that can be performed by the different workflows available in the ngs-preprocess pipeline.

The ngs-preprocess pipeline can perform several quality-control steps required for Next-Generation Sequencing (NGS) data assessment. Short or long sequencing reads can be used as input data, and the subsequent steps are determined automatically by the read type or via user configuration settings. These include contamination checking, quality trimming, adapter removal, demultiplexing, file conversion, and graphical report generation ( Figure 2). The pipeline accepts data from local storage or deposited in the public repository Sequence Read Archive (SRA). When a list of SRA IDs is given, raw sequencing data (in fastq format) will be automatically downloaded using the entrez-direct and sra-tools tools.

The following steps in the pipeline will be handled automatically based on the sequencing technology indicated in the configuration file. For sample demultiplexing, Porechop ( Wick et al., 2017a) is used for Oxford Nanopore Technologies (ONT) reads and lima for PacBio reads. Long-read qualities and statistics are evaluated, and a quality report is generated using NanoPack ( Coster et al., 2018). PycoQC ( Leger and Leonardi, 2019) can also be used to perform quality control checks on ONT reads. The program fastp ( Chen et al., 2018) is used for preprocessing short sequencing reads, including quality assessment, adapter sequence removal, trimming, and reporting. Tools included in the pipeline are summarized in Table 1.

**Table 1.** Tools included as part of the ngs-preprocess pipeline (v2.6).

Software	Function	Source code	Version
sra-tools	Data download	github.com/ncbi/sra-tools	3.0.3
entrez-direct	Data download	ncbi.nlm.nih.gov/books/NBK179288/	16.2
fastp	Short-reads processing and reports	github.com/OpenGene/fastp	0.23.2
Porechop	Long-reads processing	github.com/rrwick/Porechop	0.2.4
PycoQC	Nanopore reads reports	github.com/a-slide/pycoQC	2.5.0.3
NanoPack	Long-reads quality control	github.com/wdecoster/nanopack	1.41.0
bam2fastx	Convert PacBio BAM to FASTq	github.com/PacificBiosciences/pbtk	3.1.0
bax2bam	Convert Legacy PacBio to BAM	anaconda.org/bioconda/bax2bam	0.0.9
lima	Demultiplex	github.com/PacificBiosciences/barcoding	2.7.1
ccs	Generate PacBio HiFi	ccs.how	6.4.0

### The assembly pipeline

**Figure 3.**
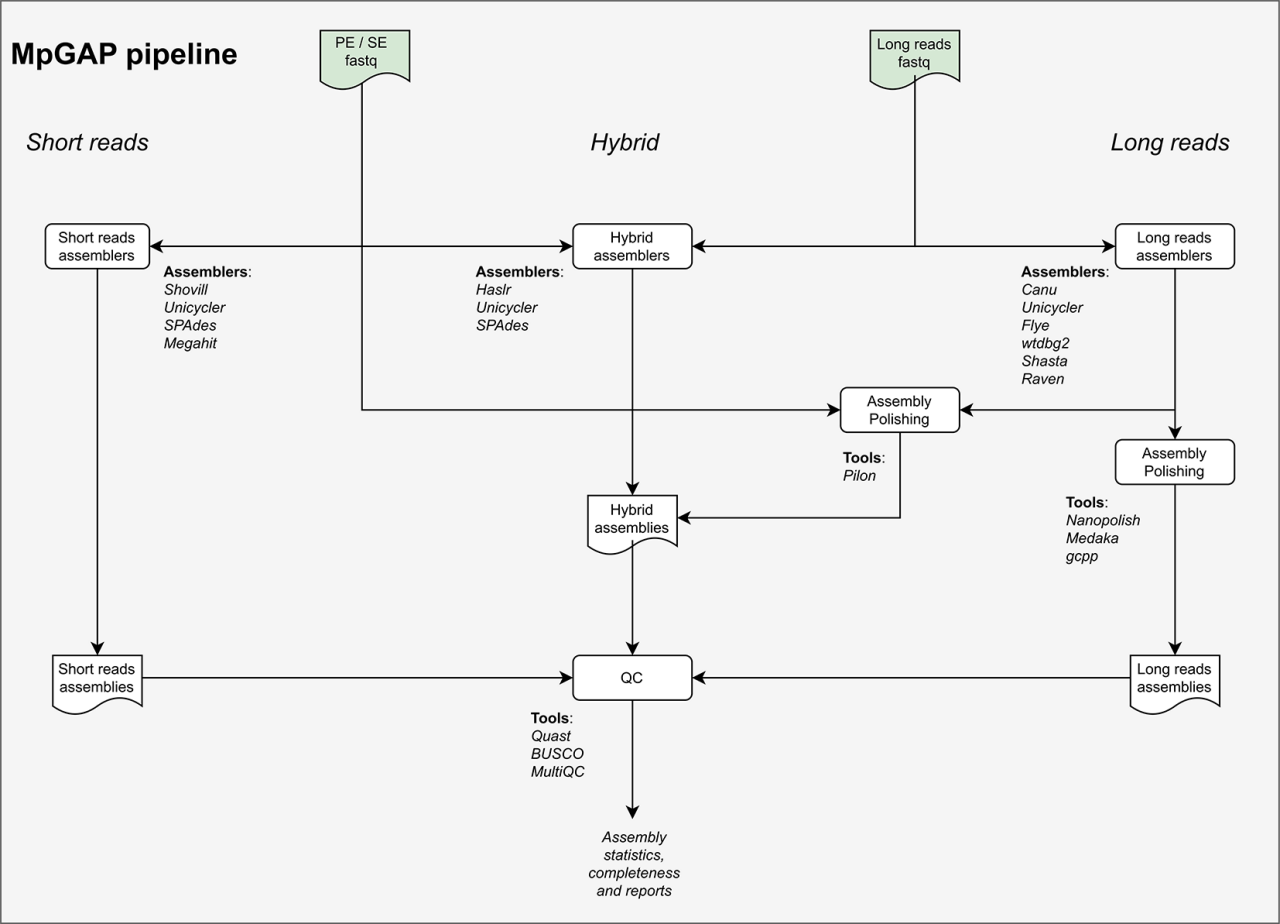
Flowchart of all steps performed by the different workflows available in the MpGAP pipeline.

The MpGAP pipeline for *de novo* genome assembly has been designed in an organism and platform-independent manner to perform short or long-read only assemblies, as well as hybrid assemblies using a combination of sequencing technologies ( Figure 3). Given the user input, the pipeline automatically selects the assembly mode. When using only short-reads, it performs the genome assembly using any of the programs: SPAdes ( Bankevich et al., 2012), Unicycler ( Wick et al., 2017b), Shovill and Megahit ( Li et al., 2015). On the other hand, when only using long-reads, it uses one or more of the following assemblers: Unicycler, Canu ( Koren et al., 2017), Flye ( Kolmogorov et al., 2019), Raven ( Vaser and Šikić, 2021), Shasta ( Shafin et al., 2020), and Wtdbg2 ( Ruan and Li, 2019). When both short and long-reads are available, the pipeline is capable of performing two types of hybrid assemblies: (1) A direct hybrid assembly using both short and long-reads sets using HASLR ( Haghshenas et al., 2020), SPAdes and Unicycler hybrid assembly modes and (2) A hybrid assembly methodology where long-reads only assembly is produced with one of the assemblers above followed by an error-correction procedure (polishing) using the available short-reads.

MpGAP is capable of polishing long-reads only assemblies using the appropriate tool based on user input: (1) Pilon ( Walker et al., 2014) for polishing with short-reads data; (2) Medaka and Nanopolish ( Loman et al., 2015) for polishing with nanopore data and (3) GCpp for polishing with PacBio data. Ultimately, assembly statistics are assessed using QUAST ( Gurevich et al., 2013) and summarized by MultiQC ( Ewels et al., 2016). All the tools that are part of the pipeline are outlined in Table 2.

**Table 2.** MpGAP pipeline core software components.

Software	Function	Source code	Version
SPAdes	Assembler	github.com/ablab/spades	3.15.3
Unicycler	Assembler	github.com/rrwick/Unicycler	0.4.8
Shovill	Assembler	github.com/tseemann/shovill	1.1.0
Megahit	Assembler	github.com/voutcn/megahit	1.2.9
Haslr	Assembler	github.com/vpc-ccg/haslr	0.8a1
Canu	Assembler	github.com/marbl/canu	2.2
Flye	Assembler	github.com/fenderglass/Flye	2.9
Raven	Assembler	github.com/lbcb-sci/raven	1.6.1
Shasta	Assembler	github.com/chanzuckerberg/shasta	0.8.0
Wtdbg2	Assembler	github.com/ruanjue/wtdbg2	2.5
Pilon	Error correction	github.com/broadinstitute/pilon	1.24
Nanopolish	Error correction	github.com/jts/nanopolish	0.13.2
Medaka	Error correction	github.com/nanoporetech/medaka	1.4.0
Gcpp	Error correction	github.com/PacificBiosciences/gcpp	2.0.2
Quast	Quality control	github.com/ablab/quast	5.0.2
MultiQC	Summary report	github.com/ewels/MultiQC	1.11

### The annotation pipeline

The bacannot pipeline is dedicated to annotating prokaryotic genomes. It covers gene prediction, annotation of gene families, mobile genetic elements, and identification of medically relevant features. The pipeline generates dynamic annotation reports and facilitates the navigation of annotated features through a genome browser.

**Figure 4.**
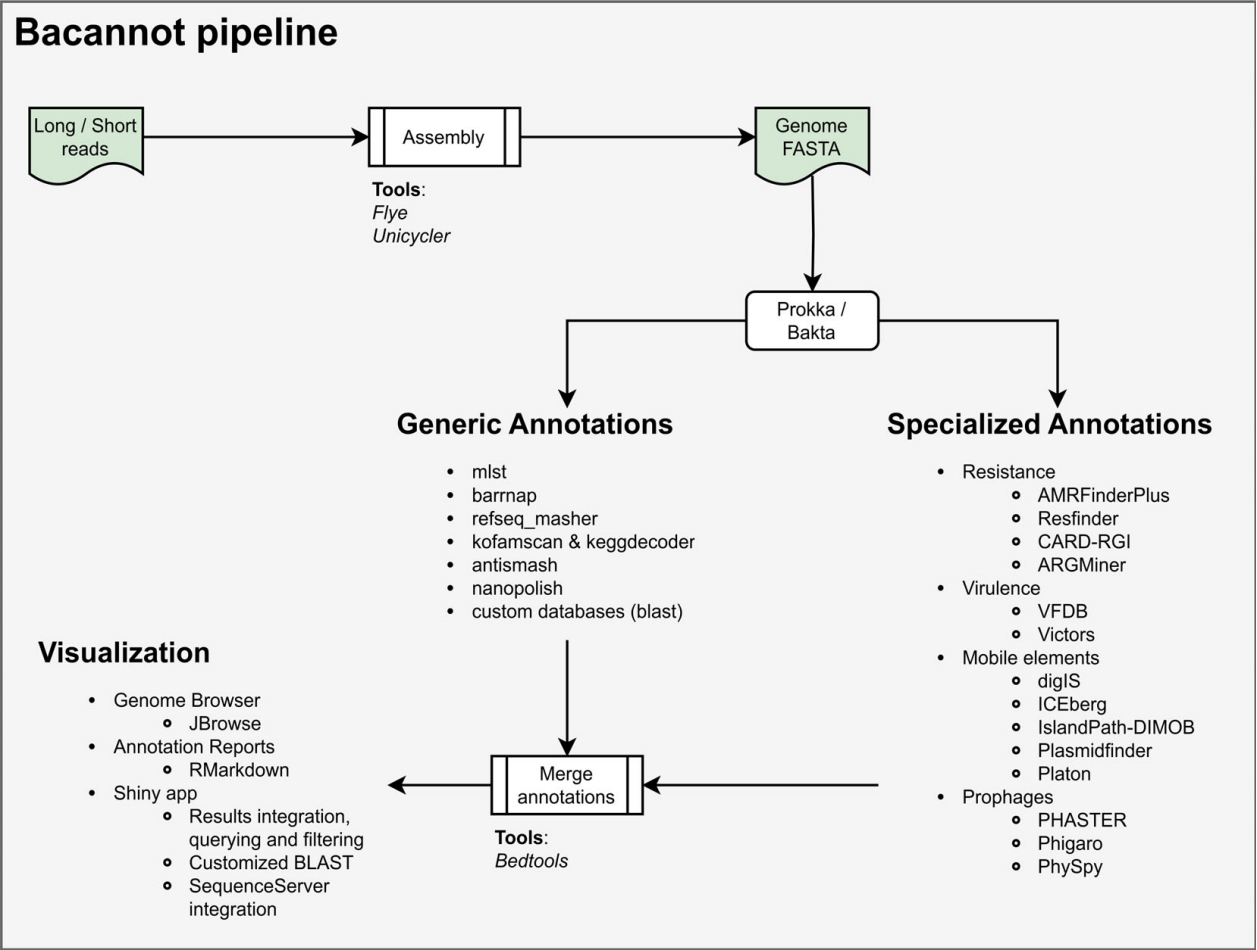
Flowchart of all analytical steps available in the bacannot pipeline.

The bacannot workflow is summarized in Figure 4. The pipeline needs a sample sheet file to initiate the process. This file should contain information about the input files, specifying whether they have preprocessed sequencing reads or assembled genomes. If the sample comprises sequencing reads, the pipeline will utilize either Unicycler ( Wick et al., 2017b) for short-reads or hybrid assembly or Flye ( Kolmogorov et al., 2019) for long-reads assembly.

The process starts with a generic genome annotation using Prokka ( Seemann, 2014) or Bakta ( Schwengers et al., 2021), followed by the prediction of rRNA sequences using barrnap, identification of closest NCBI RefSeq genome with RefSeq Masher and Multilocus Sequence Type (MLST) assignment with mlst package. Subsequently, plasmid replicons are annotated using the Plasmidfinder ( Carattoli et al., 2014) and Platon ( Schwengers et al., 2020a) software. Antimicrobial resistance genes are predicted with AMRFinderPlus ( Feldgarden et al., 2019), CARD-RGI ( Jia et al., 2017), ARGMiner database ( Arango-Argoty et al., 2020), and Resfinder ( Bortolaia et al., 2020). Virulence genes are annotated with the Virulence Factor Database (VFDB) ( Liu et al., 2018) and Victors ( Sayers et al., 2018) databases. Prophages are predicted by Phigaro ( Starikova et al., 2019), PhiSpy ( Akhter et al., 2012; Edwards et al., 2019), and PHASTER database ( Arndt et al., 2016). Genomic islands are predicted with IslandPath-DIMOB ( Bertelli and Brinkman, 2018) and plotted with gff-toolbox. Insertion sequences and integrative and conjugative elements (ICEs) are annotated with the ICEberg database ( Liu et al., 2018) and the digIS software ( Puterová and Martínek, 2021). Orthologs are assigned using KofamScan ( Aramaki et al., 2019) and visualized with KEGGDecoder ( Graham et al., 2018). Secondary metabolites are annotated with AntiSMASH ( Blin et al., 2021). Optionally, users can provide custom databases (with genes of interest) in FASTA files for additional targeted annotations with BLAST+ ( Camacho et al., 2009).

The main characteristic of this pipeline is that it is built to aid users less familiar with the tools to investigate and interpret its results with graphically rich reports. The pipeline generates HTML reports summarizing all the results using customizable document templates written in RMarkdown ( Xie et al., 2020). A genome browser created with JBrowse ( Buels et al., 2016) is also available to explore the annotated features. A custom web application has been developed using the R Shiny framework ( Chang et al., 2023) to facilitate further results exploration. It offers users additional features such as dynamic annotation filtering, as well as a built-in sequence similarity search (BLAST) functionality with an interface for executing and visualizing the results produced by SequenceServer ( Priyam et al., 2019). The software and databases used in the pipeline are listed in Table 3.

**Table 3.** Software and databases that have been made part of the bacannot pipeline (v3.2).

Software and Databases	Function	Source code	Version
Unicycler	Assembler	github.com/rrwick/Unicycler	0.4.8
Flye	Assembler	github.com/fenderglass/Flye	2.9
Prokka	Generic annotation	github.com/tseemann/prokka	1.14.6
Bakta	Generic annotation	github.com/oschwengers/bakta	1.6.1
barrnap	rRNA annotation	github.com/tseemann/barrnap	0.9
RefSeq Masher	Find closest reference	github.com/phac-nml/refseq_masher	0.1.2
mlst	Multi-Locus Sequence Typing	github.com/tseemann/mlst	2.22.1
KofamScan	Orthologs annotation	github.com/takaram/kofam_scan	1.3.0
KEEGDecoder	Pathways visualization	github.com/bjtully/BioData	1.3
Nanopolish	Methylation annotation	github.com/jts/nanopolish	0.13.2
bedtools	Data summarization	bedtools.readthedocs.io/en/latest	2.30
gff-toolbox	Data summarization	github.com/fmalmeida/gff-toolbox	0.3
AMRFinderPlus	Resistance annotation	github.com/ncbi/amr/wiki	3.10.30
ARGMiner	Resistance annotation	bench.cs.vt.edu/argminer	-
Resfinder	Resistance annotation	cge.cbs.dtu.dk/services/ResFinder	4.1
CARD-RGI	Resistance annotation	github.com/arpcard/rgi	5.2.1
PHASTER	Prophage annotation	phaster.ca	-
Phigaro	Prophage annotation	github.com/bobeobibo/phigaro	2.3.0
PhySpy	Prophage annotation	github.com/linsalrob/PhiSpy	4.2.21
IslandPath-DIMOB	Genomic Islands prediction	github.com/brinkmanlab/islandpath	1.0.6
Plasmidfinder	Plasmid detection	cge.cbs.dtu.dk/services/PlasmidFinder	2.1.6
Platon	Plasmid detection	github.com/oschwengers/platon	1.6
ICEberg	Integrative and Conjugative elements annotation	db-mml.sjtu.edu.cn/ICEberg	-
digIS	Integrative sequences detection	github.com/janka2012/digIS	1.2
Victors	Virulence annotation	phidias.us/victors	-
VFDB	Virulence annotation	mgc.ac.cn/VFs/main.htm	-
AntiSMASH	Secondary metabolites annotation	antismash.secondarymetabolites.org/	6.1.1
JBrowse	Results visualization	jbrowse.org/jbrowse1.html	1.16.9
SequenceServer	BLAST visualization	https://sequenceserver.com/	2.0.0

### Biological sample, DNA extraction, susceptibility test, and sequencing

The sample KpBSB53 was collected at the University Hospital of Brasilia, Brazil, in April 2016 from the tracheal aspirate of a 41-year-old man. The VITEK 2 system (BioMérieux) was used for microbial identification. Antibiotic susceptibility was tested by the disk diffusion method as described by de Campos et al. (2021). The following antibiotics were tested: Amikacin, Aztreonam, Cefepime, Ceftazidime, Ciprofloxacin, Gentamicin, Imipenem, Levofloxacin, Meropenem, Norfloxacin, Ofloxacin, Piperacillin/tazobactam, Polymyxin, Tobramycin, Ticarcillin/clavulanate (Sensidisc DME - Diagnósticos Microbiológicos Especializados). After 24 h of incubation at 37°C, the sample was classified as susceptible, resistant multiresistant based on what was described by the manufacturer. DNA extraction was performed as described by Ausubel et al. (1992), and extracted DNA was quantified with Nanodrop™. DNA sequencing was performed with both long and short-read technologies. Long-read sequencing was performed using an Oxford Nanopore Technologies MinION Mk1b device, using the rapid barcode kit (SQK-RBK-004) in a R9.4.1 SpotON *flowcell* (FLO-MIN106D). Short-read sequencing was performed by BGI (Shenzhen, China) using paired-end reads with 150 bp on a DNBseq ^®^ platform.

### Computational analyses

For the processing of the bacterial genome above, the raw long and short-reads have been analyzed sequentially with the developed pipelines. Quality assessment, filtering, and trimming were performed using the ngs-preprocess pipeline v2.6. In our use case, aside from the default parameters, we set the pipeline to correct the short paired-end reads with fastp and filter long-reads based on quality ( ≥ 10) and length ( ≥ 750) using the parameters *--lreads_min_length* and *--lreads_min_quality*, respectively.

The preprocessed sequencing reads were assembled using the MpGAP pipeline v3.1.4. Genome assembly was performed using the hybrid method where long-reads are first assembled with long-reads assemblers and afterward polished using the short-reads data ( *--hybrid_strategy* 2). The hybrid assembly strategy was executed only with the Flye assembler ( Kolmogorov et al., 2019) instead of using all the available options to limit the computational burden. The BUSCO ( Simão et al., 2015) completion assessment was performed with the bacteria_odb9 dataset, containing 148 expected bacterial genes.

The final polished genomes were annotated using the bacannot pipeline v3.1.5. The pipeline’s required databases, such as VFDB ( Liu et al., 2018), were downloaded in May 2022. The parameter *--resfinder_species* has been set to “ *Klebsiella*”. We used Prokka ( Seemann, 2014) for generic annotation, and the tool’s database was enhanced with the public NCBI Prokaryotic Genome Annotation Pipeline (PGAP) HMM library ( Li et al., 2020) by using the *--prokka_use_pgap* parameter.

### Operation

The pipeline requires POSIX-compliant systems (e.g., Linux or OS X) or Windows with Windows Subsystem for Linux (WSL2). Pre-installation requirements on the executing computer are Nextflow, and a software management tool (Conda) or a container platform, either Docker or Singularity. Each pipeline requires a configuration file describing the samples and corresponding data to be used. These files also set the specific software to be used in pipeline steps with numerous alternatives (e.g., assembler program) and execution parameters for the whole pipeline or specific software. The configuration files are text files (in YAML format) that should be modified by the user.

With all these requirements satisfied, the execution is conducted non-graphically in a terminal. For example, to trigger the sequential execution of the pipelines, the following command lines should be issued sequentially:

nextflow run fmalmeida/ngs-preprocess -profile docker -latest -params-file preprocess-params.yml

nextflow run fmalmeida/mpgap -profile docker -latest -params-file assembly-params.yml

nextflow run fmalmeida/bacannot -profile docker -latest -params-file annotation-params.yml

The exemplified command lines would trigger the execution of the latest version of the pipeline’s code using docker as the container engine, with all the required parameters configured in the YAML file. Optionally, it is possible to launch specific versions of the pipeline for reproducibility using the “-r” parameter, e.g., “-r v3.2”.

In order for users to replicate the analyses of this paper, the configuration files and command lines executed have been made available in a Zenodo project. The configuration file and the sample sheet used for the annotation pipeline have been provided as Supplementary Material (1 and 2) for quick visualization of the expected format. All pipelines have a complete description of workflows and input files in the online manuals.

## Results

This paper showcases the utilization of the developed pipelines to conduct a thorough genome examination of an unpublished bacterial sample sequenced with both short and long-reads. The sample, namely KpBSB53 (see Methods), microbiologically classified as *Klebsiella pneumoniae*, was isolated from the tracheal aspirate of a 41-year-old man at the University Hospital of Brasilia. The isolate was susceptible to all the antibiotics tested.

The pipeline assembles and annotates genes for prokaryotes and performs additional annotation steps when examining a clinical sample. This feature makes the tool especially useful for investigating antimicrobial resistance. Using a clinical sample exposes additional tasks included in the pipeline that apply to antimicrobial resistance studies. Subsequently, the KpBSB53 sample will be used to demonstrate the operation of the pipelines to analyze a clinically relevant bacterial genome, as well as a description of the generated results and how to interpret them.

### Reads preprocessing and quality control

The process begins with the ngs-preprocess pipeline, which carries out quality control and cleaning actions essential for subsequent genome assembly and annotation tasks. Additionally, it offers the user an important assessment of the sequencing outcomes of the samples.

The output of the ngs-preprocess pipeline is a directory containing the preprocessed sequencing reads organized according to the sequencing technology of the input files, as indicated in the user configuration file. The preprocessed reads can have sequencing adapter or low-quality stretches removed (trimming) or be discarded entirely if a minimum number of good-quality bases is not reached. The pipeline also provides plots and reports for quality control (QC) inspection.

**Figure 5.**
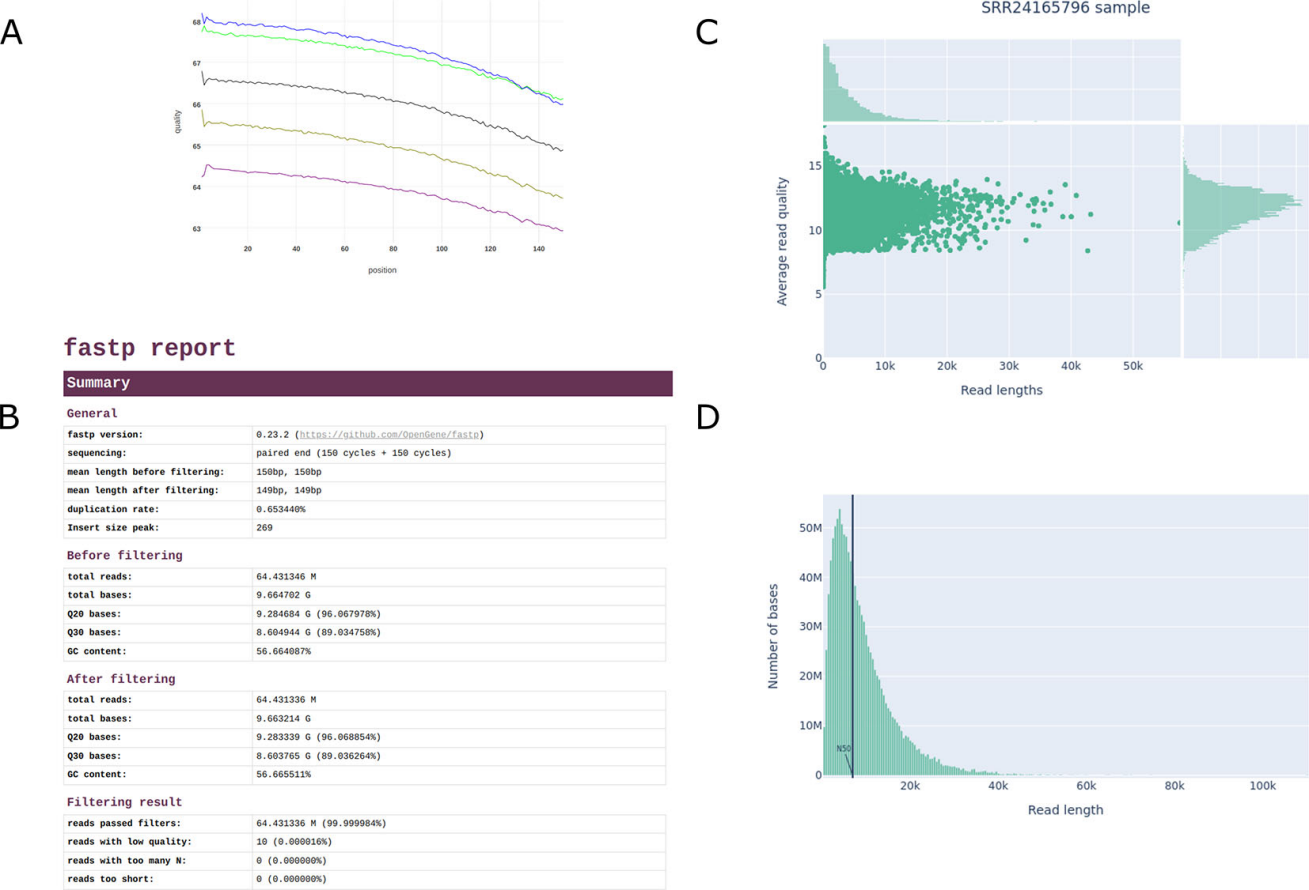
Overview of typical QC outputs generated by the ngs-preprocess pipeline. Figures A and B are generated by the fastp tool and display the base quality of one of the short-read pairs and the summary of reads statistics, respectively. Figures C and D are generated by the NanoPlot tool and display the average read quality per read length and the weighted read length histogram, respectively.

In the particular case of the KpBSB53 sample, the short-reads obtained were of outstanding quality, displaying quality values ≥ 60 before preprocessing ( Figure 5A and B). The nanopore data fluctuated around the expected average quality and length, with median values of ≈ 10 and ≈ 4 kb, respectively ( Figure 5C and D).

### Genome assembly

After running ngs-preprocess pipeline, users can evaluate the quality of the input sequencing reads and ensure that only data with enough quality will be used for genome assembly. Once selected, the user manually supplies the preprocessed sequencing reads (such as short-reads, Nanopore, or PacBio) to the MpGAP pipeline using a sample sheet in a text file (see Methods), allowing users to choose from an assortment of assembly programs and strategies to perform the assembly. The pipeline will automatically select the appropriate assembly program based on the sequencing reads provided.

The KpBSB53 sample illustrates the pipeline’s versatility and ability to work with both short and long-reads. For this sample, the Flye assembler ( Kolmogorov et al., 2019) was chosen for a hybrid assembly approach ( Chen et al., 2020), which initially has errors owing to sequencing technology, later corrected by including short-read data (polishing) using Pilon ( Walker et al., 2014).

The pipeline generates a sub-directory for each sample containing the assembly results, including the initial and polished assembly files, and creates a report using MultiQC ( Ewels et al., 2016) that includes assembly quality metrics from Quast ( Gurevich et al., 2013) and BUSCO ( Simão et al., 2015) to facilitate comparison between the assemblies. The user can choose different programs to analyze the same data, and report files are available to aid in selecting the best assembly.

After analyzing the KpBSB53 sample with this pipeline, we found that it consists of two circular contigs - one representing a chromosome (5.2 Mb) and the other a plasmid (142 kb), essentially a full replicon resolution. The BUSCO metrics results highlight the reference quality level of this genome, with 98.65% ortholog completeness in the bacterial chromosome.

### Genome annotation

**Figure 6.**
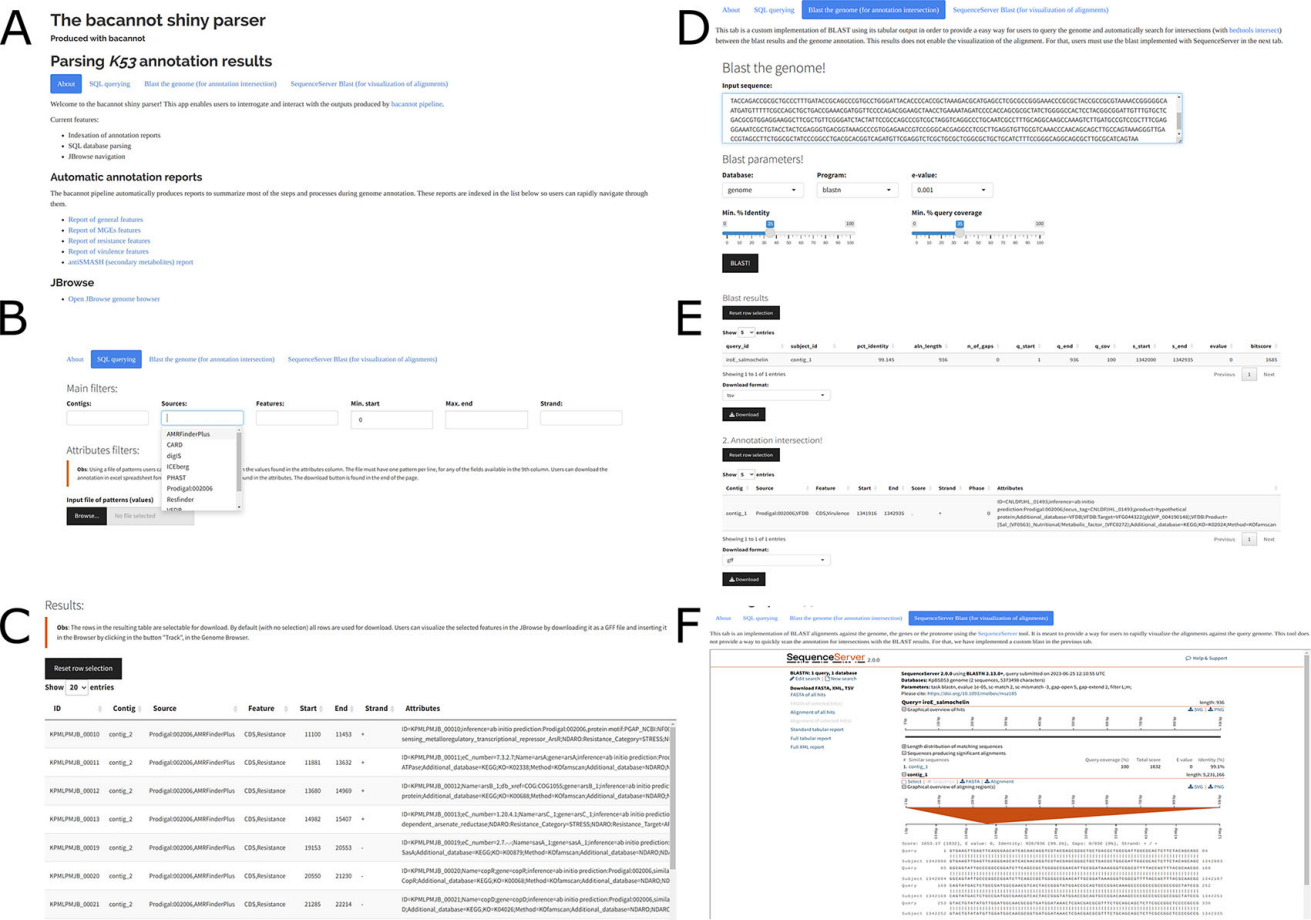
Overview of the main features available in the web-based application for results exploration made available as part of the bacannot pipeline. Figure A is the homepage of the application for navigation between available features. Figures B and C display the dynamic text-filtering of annotation results. Figures D and E exemplify the interactive results filtering and investigation based on sequence alignment. Figure F shows the SequenceServer tool included in the application for execution and visualization of BLAST alignments.

**Figure 7.**
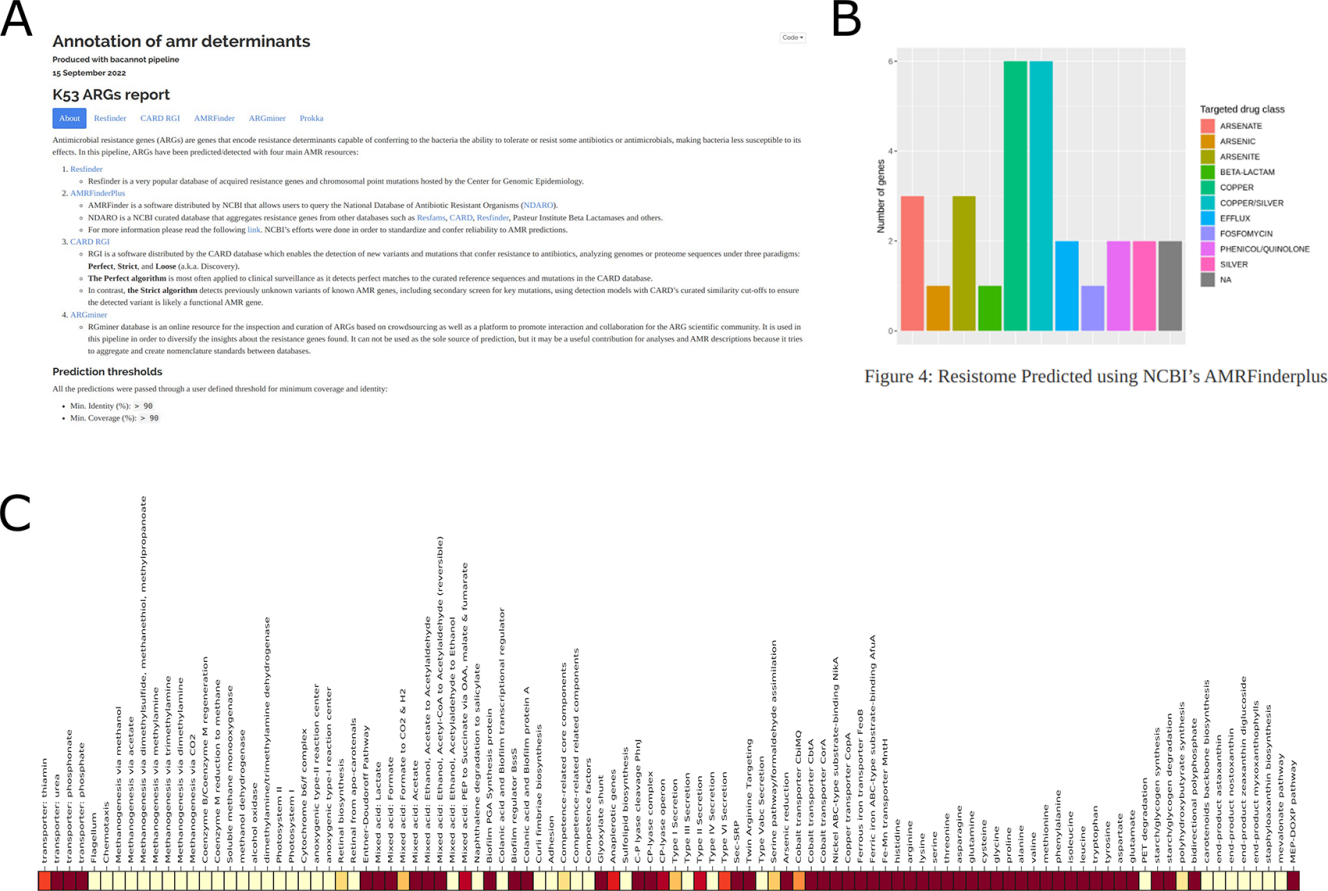
Overview of the specialized automatic HTML reports generated by the bacannot pipeline. Figures A and B are screenshots of the antimicrobial resistance (AMR) automatic report, highlighting its homepage containing the annotation description and summary along with a bar plot displaying all the features annotated by the AMRFinderPlus tool. Figure C shows a partial screenshot of the KEGG annotation heatmap autogenerated using KOfamscan and KEGGDecoder tools.

**Figure 8.**
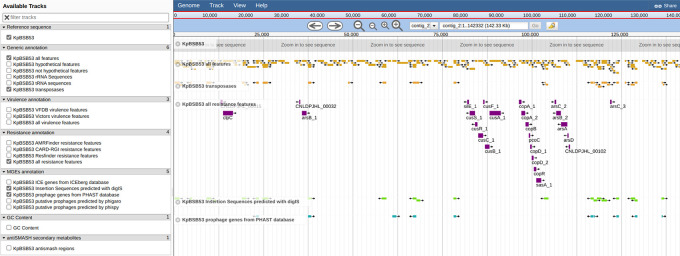
Overview of the automatically rendered genome browser using JBrowse made available as part of the bacannot outputs. For illustration, the annotation tracks showing predicted antibiotic resistance genes and insertion sequences are activated in a region of the KpBSB53 plasmid.

The assembly reports offer guidelines to assist users in choosing the most suitable assembly alternative for their sample. After making their selection, users can manually indicate the chosen assembly results to the annotation pipeline through the configuration file. Bacannot provides a structured and consistent output, allowing straightforward summarization and examination of its contents. Though multiple annotation stages produce outputs, the primary focus will be on the pipeline’s essential results, namely:

1. Complete genome annotation.
2. A web-based application for results visualization and exploration ( Figure 6).
3. Automatic HTML reports for resistance, virulence, mobile elements, and annotations from specialized databases ( Figure 7).
4. A genome browser for visualization of annotated features ( Figure 8).

After all the specialized programs in bacannot have finished their analysis, the results are consolidated into a single General Feature Format file (GFF) and a GenBank format file (GBK) containing the complete genome annotation. These files can be used in other general investigation programs or submitted to NCBI databases. The annotated features are also saved as nucleotide and protein sequences in FASTA format. Moreover, these files are processed internally and presented in the web application as interactive web pages ( Figure 6A). This workbench allows users to filter results by text or sequence using the SequenceServer and BLAST applications. The filtered results can be converted into tables of varying formats ( Figure 6B and C). The BLAST function included in this workbench allows users to easily annotate other target sequences even after the pipeline is finished. Users can find intersections of alignment results with the genome annotation ( Figure 6E and E) or visualize alignments with SequenceServer ( Figure 6F).

Moreover, detailed reports are generated for feature-specific analyses, such as antibiotic gene prediction, consolidating the results, and providing cross-references to the source databases used for annotation ( Figure 7). Besides these clinically-relevant features, the pipeline also has other generic annotation modules that are useful for any bacterial strain, such as prophage and secondary metabolites annotation, KEGG KO annotation, and the possibility of using custom annotation databases or a list of NCBI protein IDs. The KO module, for instance, outputs a text file ready for the KEGG mapper tool to generate pathway figures.

The genome browser allows users to visually explore the annotation results and investigate the genomic context of relevant genes. The browser contains various specialized tracks for the targeted annotations performed by the pipeline, such as for resistance, virulence, prophages, and more. For example, in Figure 8, one can quickly observe a cassette of stress-related resistance genes surrounded by insertion sequences in the plasmid contig.

One of the first results generated by this pipeline is the strain classification based on the alignment-free sequence distance against a database of bacterial genomes (NCBI RefSeq) as calculated by RefSeq Masher. The strain BIDMC 55 (GCF_000692955.1) isolated in the USA was the closest genome to strain KpBSB53 (Mash distance=0.000715202). Additionally, an *in silico* multilocus sequence typing (MLST) is executed using the mlst program against the BIGSdb PubMLST database ( Jolley and Maiden, 2010), revealing that our strain belongs to the *Klebsiella pneumoniae* ST 105 group, which has been reported as the driver of a plasmid-mediated outbreak of NDM-1-producing strains in China ( Zheng et al., 2016). Using Prokka ( Seemann, 2014) for generic annotation, 4937 coding sequences (CDS) have been detected, alongside 25 rRNAs and 87 tRNAs. Another pipeline result is the prediction of plasmid replicons using PlasmidFinder ( Carattoli et al., 2014). For the KpBSB53 strain, a single plasmid was predicted and classified as IncFIB(K), a very dynamic replicon that is mostly associated with MDR plasmids in *K. pneumoniae* ( Lahlaoui et al., 2015; Tian et al., 2021) and important for virulence as well ( Tian et al., 2021).

The pipeline includes several analyses for annotating antimicrobial resistance genes as default. Strain KpBSB53 has only a few acquired antibiotic-resistance genes, namely *blashv-*1, *fosA*, and *oqxAB*, all considered intrinsic to the species and located in the chromosome ( Bernardini et al., 2019; Holt et al., 2015). This agrees with our experimental results, which show that this strain is susceptible to all antibiotic classes tested.

Additionally, a set of stress-related genes (Supplementary Material 3) conferring resistance to copper, silver, and other metalloids have been detected in the plasmid sequence ( Figure 7). On the other hand, several virulence genes have been detected in the sample’s genome using the Virulence Factor Database (VFDB) ( Liu et al., 2018). Despite the classical virulence genes normally found in *Klebsiella pneumoniae* strains ( Paczosa and Mecsas, 2016), strain KpBSB53 encodes three operons for siderophore biosynthesis: enterobactin ( *entABCDEFS*, *fesABCDG*), salmochelin ( *iroE*) and aerobactin ( *iutA*). Moreover, the type 1 and 3 fimbriae and an *ecp* ( *E. coli* common pilus) gene have also been detected. These three fimbriae types are directly related to the adhesion to surfaces, interaction with host cells, and biofilm formation ( Alcántar-Curiel et al., 2013). Taken together, the presence of several virulence factors suggests that the KpBSB53 sample may be a hypervirulent strain or at least more virulent than the classical *K. pneumoniae* strains, as discussed by Paczosa and Mecsas (2016).

### Resources usage

Table 4 presents the expected computer resources and timings for the execution of all pipelines on a standard Linux laptop. A Linux Ubuntu 22.04 laptop, with 4 CPUs (8 cores) and 18 Gb RAM, was used in this study. The computational requirements of the ngs-preprocess pipeline are low. However, the time it takes to finish is positively related to the amount of data. A single tool called fastp is used for short-read preprocessing, which takes around 7 minutes to complete and requires approximately 3 Gb of RAM. For nanopore reads, porechop is the most resource-intensive module, taking approximately 1 hour to finish and using approximately 5 Gb of RAM. In terms of the MpGAP assembly pipeline, the execution depth highly depends on the amount of sequencing data provided and is the most resource-intensive step in the workflow. In our hybrid assembly use case, it accounts for half of the overall processing time and requires 11 Gb of RAM ( Table 4).

**Table 4.** Resource usage metrics for the execution of all pipelines using strain KpBSB53 data, as measured automatically by Nextflow. For bacannot, only the most time and memory-consuming tasks are shown.

Pipeline	Software	Task	Duration (min)	% of pipeline duration	Memory (Gb)
ngs-preprocess	fastp	short-reads preprocessing	7	7.6	2.7
ngs-preprocess	porechop	long-reads preprocessing	70	76.1	4.4
ngs-preprocess	nanopack	filter and quality check	15	16.3	2.1
MpGAP	Flye	assembly	48	5.4	11.3
MpGAP	medaka	long-reads polishing	28	3.18	9.5
MpGAP	pilon	short-reads polishing	625	70.8	10.6
MpGAP	QUAST & Busco	quality check	182	20.6	10.2
MpGAP	MultiQC	reporting	0.2	0.02	0.7
bacannot	Prokka	gene annotation	16	43.2	1
bacannot	Platon	plasmid detection	2	5.4	3.6

## Discussion

In the last decade, advances in DNA sequence generation resulted in a steadfast incorporation of genomics into routine microbiological research practice, ranging from clinical to environmental applications ( Didelot and Parkhill, 2022). Despite the significant progress made by the establishment of multiple computational protocols to process genomic data, a considerable gap still needs to be bridged for these to be readily integrated into laboratory practices. The primary reason behind the problem is the lack of necessary bioinformatics foundations, including a shortage of skilled personnel or proper infrastructure.

Our belief is that genomics pipelines should possess certain inherent characteristics to promote their widespread use. These attributes include easy installation and execution, modularity and extensibility, and the generation of user-friendly reports that consolidate the results and foster biological interpretation.

Considering these tenets, we have created a comprehensive set of bacterial genomics pipelines ( ngs-preprocess, MpGAP, and bacannot). These specialized pipelines should be invoked in sequence, taking raw data from any sequencing platform and converting them into an annotated genome, emphasizing antimicrobial resistance and virulence genes. The first two modules are not restricted to bacterial data, making it possible to analyze data from other organisms. Conversely, the bacannot pipeline is specific to bacteria but can accept sequencing reads as input, allowing users to assemble and annotate their genome with a single command, which is particularly helpful for those less experienced with bioinformatics.

By following a few simple steps (available online), users can customize the execution of these pipelines and obtain complete results in under a day using a commodity computer. These pipelines utilize technology for modular workflow composition, installation, and execution through virtual containers that package all necessary software requirements. This modularity enables incremental updates in response to community requirements which are transparently pushed to the users unless a specific version is requested for reproducibility purposes.

Currently, we are aware of six established bacterial genomics pipelines that are actively maintained and have comparable characteristics to our three pipelines. These include ASA3P ( Schwengers et al., 2020b), TORMES ( Quijada et al., 2019), Nullarbor, Bactopia ( Petit and Read, 2020), MicrobeAnnotator ( Ruiz-Perez et al., 2021) and MicroPIPE ( Murigneux et al., 2021). Although they have some common elements and comparable assembly and annotation modules, each software has unique features tailored for specific purposes. Their goals and designs vary, providing users with high-quality options for various analytical scenarios. Table 5 outlines the differences in capabilities among the annotation pipelines. We now highlight some of the distinctive features of these pipelines.

**Table 5.** Comparison of selected bacterial genome annotation pipelines.

Feature	Bacannot	Bactopia	ASA3P	MicrobeAnnotator	Nullarbor	TORMES
Sequence technology	Short and Long-reads	Short and Long-reads	Short and Long-reads	Short-reads	Short-reads	
Assembly type	Short-reads, Long-reads and Hybrid	Short-reads and Hybrid	Short-reads, Long-reads and Hybrid	Paired-end short-reads only	Paired-end short-reads only	
Single-end reads	Yes	Yes	Yes	No	No	
Workflow	Nextflow	Nextflow	Groovy	Python	Perl + Make	Bash
Resume if stopped	Yes	Yes	No	Yes	Yes	No
Computing cluster and cloud compatibility	Yes	Yes	Yes	No	No	No
Batch processing from config file	Yes	Yes	Yes	Yes	Yes	Yes
Summary reports	HTML, Genome Browser and Shiny Application	Text	HTML	Text and Image	HTML	R Markdown
Package manager	Nextflow + Github	Nextflow + Conda	Conda	Conda and Brew	Conda	
Container available	Yes	Yes	Yes	No	No	No
Documentation	Website and Readme	Website	PDF Manual and Readme	Readme	Readme	Readme
Source code	https://github.com/fmalmeida/bacannot	https://github.com/bactopia/bactopia	https://github.com/oschwengers/asap	https://github.com/cruizperez/MicrobeAnnotator	https://github.com/tseemann/nullarbor	https://github.com/nmquijada/tormes
Latest release (version/year)	v3.2/2022	v2.2.0/2022	v1.3.0/2020	v2.0.5/2021	v2.0.20191013/2019	v1.3.0/2021

Our three pipelines provide a comprehensive bacterial genomics analysis from unprocessed reads to annotation similar to what ASA3P, TORMES, Nullarbor, and Bactopia offer. The analysis provided by MicroPIPE and MicrobeAnnotator is less comprehensive than the other pipelines. MicroPIPE is crafted for genome assembly and covers the process from base-calling to genome polishing. However, our MpGAP genome assembly pipeline is more versatile as it can accommodate data from Illumina, PacBio, and ONT sequencing technologies and has nine assemblers with distinctive assembly strategies. This flexibility enables users to select the most suitable choice for their requirements. MicrobeAnnotator is a pipeline that specializes in functional genome annotation. It offers a KEGG Orthology annotation step through Kofamscan, also available in bacannot. However, MicrobeAnnotator only focuses on the functional annotation of predicted genes using KEGG, UniProt, RefSeq, and Trembl. Unlike other pipelines, it does not include extra modules like virulence and resistance gene annotation.

Analogous to Bactopia, our pipelines are very flexible and customizable. Compared to bacannot, Bactopia has more functionalities but cannot currently produce visual reports for results inspection. Moreover, those with limited bioinformatics experience may find the additional configuration steps more complex. Additionally, Bactopia provides extensions to its core workflow with post-processing tools intended, for example, for pangenome and phylogenetic analyses. Bacannot does not offer these as default, but the standardization of its outputs enables users to adapt them for such tasks.

Bacannot stands out for its added ability to annotate various genomic feature classes as part of its central workflow, including secondary metabolites, prophages, genomic islands, integrative and conjugative elements (ICEs), and DNA methylation, without requiring additional executions. This analytic range permits the annotation of clinically relevant traits and provides valuable attributes for non-clinical samples. As a result, bacannot has been used in various scenarios, including the analysis of clinical samples ( de Campos et al., 2021), environmental samples from a lake ( Janssen et al., 2021), soil ( Belmok et al., 2023), and plant-associated bacteria ( Bartoli et al., 2022; Ramírez-Sánchez et al., 2022).

Lastly, bacannot is the only tool that includes a comprehensive set of dynamic reports, presented as a built-in web application, along with a genome browser, offering a unified and interactive platform for interrogation and visualization of the annotation results.

Using the developed pipelines, we analyzed a strain of *Klebsiella pneumoniae* (KpBSB53) isolated from a patient at the University Hospital of Brasilia. We aimed to characterize its antibiotic resistance profile and virulence at the genome sequence level. We used sequencing data from short and long-read platforms to conduct a comprehensive genomic analysis of the strain on a laptop with 18 Gb of memory in less than a day. Our findings revealed that the strain belongs to the ST 105 group, associated with a neonatal unit outbreak in China ( Zheng et al., 2016). The annotation of resistance genes only identified components considered intrinsic to the species ( Bernardini et al., 2019; Holt et al., 2015). Additionally, mutations in *acrR*, *ompK36*, and *ompK37* genes were found, which may play a role in resistance. These findings are consistent with the experimentally observed susceptibility to the tested antibiotics.

## Conclusions

This work showcases three bioinformatics pipelines that, together, provide a complete workflow for a thorough analysis of bacterial genomics using next-generation sequencing data. These computational protocols encompass the entire process, from the initial raw reads to the final genome assembly and gene annotation. It is recommended to execute them in succession, but they can also function independently by incorporating external data provided by the user.

The pipelines were designed to simplify the installation process by incorporating the many specialized software tools required for all stages in the form of virtualized containers. This eliminates the complexity of setup tasks, allowing the pipelines to be deployed and executed with a single command line. We also provide thorough documentation to expand the user base and make them accessible to those without bioinformatics expertise.

We not only focused on processing data but also on creating graphical reports and visualizations to improve result interpretation. To achieve this, we developed a web-based tool that allows users to analyze and refine the results using text or sequence annotation, distinguishing bacannot from other pipelines.

The existence of several comparable pipelines indicates that there is no one-size-fits-all approach to genomics. Generating DNA sequence data is becoming more widespread, but there is a significant challenge in analyzing this data. Our set of tools has been developed to address this issue and aid in the study of bacterial genomics.

## Ethical considerations

The studies involving human participants were reviewed and approved by the ethical approval received from the Faculdade de Medicina, Universidade de Brasília, Brasília, DF, Brazil (approval no. CEP/FMUnB 1.131.054; CAEE: 44867915.1.0000.558). The patients provided their written informed consent to participate in this study.

## Data Availability

Sample sequencing data has been made in NCBI, BioProject PRJNA955456, BioSample: SAMN34178607. Additionally, the code required to reproduce the analysis performed in this paper has been made available in a Zenodo repository containing bash scripts with all required configurations. Zenodo. Scalable and versatile container-based pipelines for de novo genome assembly and bacterial annotation (Sup. Material) https://doi.org/10.5281/zenodo.7859428. This project contains the following underlying data:
• input/sra_ids.txt (list of SRA ids for the preprocessing pipeline) • preprocess-params.yml (pre-set parameters file for the preprocessing pipeline) • run_preprocess.sh (script to run preprocessing pipeline with pre-set configutations) • assembly-params.yml (pre-set parameters file for the assembly pipeline) • assembly.config (pre-set resources configuration file for the assembly pipeline) • assembly_samplesheet.yml (pre-generated samplesheet for the assembly pipeline) • run_assembly.sh (script to run assembly pipeline with pre-set configuration) • annotation-params.yml (pre-set parameters file for the annotation pipeline) • annotation_samplesheet.yml (pre-generated samplesheet for the annotation pipeline) • run_annotation.sh (script to run annotation pipeline with pre-set configuration) Data are available under the terms of the Creative Commons Attribution 4.0 International license (CC-BY 4.0).

## Software availability

The pipelines are available as GitHub repositories under the GNU General Public License v3.0. The pipeline and dependencies are easily installed by combining Nextflow and Docker or Singularity, all described in the documentation. The repositories are:

• ngs-preprocess - version controlled https://github.com/fmalmeida/ngs-preprocess
• ngs-preprocess - archived, Zenodo: https://zenodo.org/record/7831610
• MpGAP - version controlled https://github.com/fmalmeida/MpGAP
• Mpgap - archived, Zenodo: https://zenodo.org/record/7046782
• bacannot - version controlled https://github.com/fmalmeida/bacannot
• bacannot - archived, Zenodo: https://zenodo.org/record/7459261
• License: GNU General Public License v3.0 only

## References

[ref67] Alcántar-Curiel MD Blackburn D Saldaña Z : Multi-functional analysis of Klebsiella pneumoniae fimbrial types in adherence and biofilm formation. *Virulence.* February 2013;4(2):129–138. 10.4161/viru.22974 23302788 PMC3654611

[ref47] Aramaki T Blanc-Mathieu R Endo H : KofamKOALA: KEGG ortholog assignment based on profile HMM and adaptive score threshold. *bioRxiv.* April 2019;602110. 10.1101/602110 PMC7141845 31742321

[ref37] Arango-Argoty GA Guron GKP Garner E : ARGminer: A web platform for the crowdsourcing-based curation of antibiotic resistance genes. *Bioinformatics.* February 2020;36(9):2966–2973. 10.1093/bioinformatics/btaa095 32058567

[ref42] Akhter S Aziz RK Edwards RA : PhiSpy: A novel algorithm for finding prophages in bacterial genomes that combines similarity- and composition-based strategies. *Nucleic Acids Res.* May 2012;40(16):e126–e126. 10.1093/nar/gks406 22584627 PMC3439882

[ref44] Arndt D Grant JR Marcu A : PHASTER: A better, faster version of the PHAST phage search tool. *Nucleic Acids Res.* May 2016;44(W1):W16–W21. 10.1093/nar/gkw387 27141966 PMC4987931

[ref56] Ausubel FM Brent R Kingston RE : *Short protocols in molecular biology.* New York:1992; vol.275:28764–28773.

[ref18] Bankevich A Nurk S Antipov D : SPAdes: A new genome assembly algorithm and its applications to single-cell sequencing. *J. Comput. Biol.* May 2012;19(5):455–477. 10665277. 10.1089/cmb.2012.0021 22506599 PMC3342519

[ref72] Belmok A Almeida FM de Rocha RT : Genomic and physiological characterization of Novosphingobium terrae sp. nov., an alphaproteobacterium isolated from Cerrado soil containing a mega-sized chromid. *Braz. J. Microbiol.* March 2023;54(1):239–258. 1517-8382, 1678-4405. 10.1007/s42770-022-00900-4 36701110 PMC9944591

[ref73] Bartoli C Rigal M Mayjonade B : Unraveling the genetic architecture of the adaptive potential of Arabidopsis thaliana to face the bacterial pathogen Pseudomonas syringae in the context of global change. *Pathology.* August 2022. Preprint.

[ref2] Berger B Yu YW : Navigating bottlenecks and trade-offs in genomic data analysis. *Nat. Rev. Genet.* December 2022;24(4):235–250. 10.1038/s41576-022-00551-z 36476810 PMC10204111

[ref45] Bertelli C Brinkman FSL : Improved genomic island predictions with IslandPath-DIMOB.In Alfonso Valencia, editor, *Bioinformatics.* July 2018;34: pp.2161–2167. 10.1093/bioinformatics/bty095 29905770 PMC6022643

[ref64] Bernardini A Cuesta T Tomás A : The intrinsic resistome of Klebsiella pneumoniae. *Int. J. Antimicrob. Agents.* January 2019;53(1):29–33. 10.1016/j.ijantimicag.2018.09.012 30236960

[ref49] Blin K Shaw S Kloosterman AM : antiSMASH 6.0: Improving cluster detection and comparison capabilities. *Nucleic Acids Res.* May 2021;49(W1):W29–W35. 10.1093/nar/gkab335 33978755 PMC8262755

[ref38] Bortolaia V Kaas RS Ruppe E : ResFinder 4.0 for predictions of phenotypes from genotypes. *J. Antimicrob. Chemother.* August 2020;75(12):3491–3500. 10.1093/jac/dkaa345 32780112 PMC7662176

[ref52] Buels R Yao E Diesh CM : JBrowse: A dynamic web platform for genome visualization and analysis. *Genome Biol.* December 2016;17(1):66. 1474760X. 10.1186/s13059-016-0924-1 27072794 PMC4830012

[ref50] Camacho C Coulouris G Avagyan V : BLAST+: Architecture and applications. *BMC Bioinformatics.* December 2009;10(1). 10.1186/1471-2105-10-421 20003500 PMC2803857

[ref55] Campos TA de Almeida FM de Almeida APC de : Multidrug-Resistant (MDR) Klebsiella variicola Strains Isolated in a Brazilian Hospital Belong to New Clones. *Front. Microbiol.* April 2021;12:604031. 1664-302X. 10.3389/fmicb.2021.604031 33935984 PMC8085564

[ref53] Chang W Joe Cheng JJ Allaire CS : Shiny: Web Application Framework for R. 2023.

[ref33] Carattoli A Zankari E García-Fernández A : In Silico Detection and Typing of Plasmids using PlasmidFinder and Plasmid Multilocus Sequence Typing. *Antimicrob. Agents Chemother.* July 2014;58(7):3895–3903. 10.1128/aac.02412-14 24777092 PMC4068535

[ref17] Chen S Zhou Y Chen Y : Fastp: An ultra-fast all-in-one FASTQ preprocessor. *Bioinformatics.* September 2018;34(17):i884–i890. 10.1093/bioinformatics/bty560 30423086 PMC6129281

[ref59] Chen Z Erickson DL Meng J : Benchmarking hybrid assembly approaches for genomic analyses of bacterial pathogens using Illumina and Oxford Nanopore sequencing. *BMC Genomics.* December 2020;21(1):631. 1471-2164. 10.1186/s12864-020-07041-8 32928108 PMC7490894

[ref15] De Coster W D’Hert S Schultz DT : NanoPack: Visualizing and processing long-read sequencing data. *Bioinformatics.* March 2018;34(15):2666–2669. 10.1093/bioinformatics/bty149 29547981 PMC6061794

[ref12] Di Tommaso P Chatzou M Floden EW : Nextflow enables reproducible computational workflows. *Nat. Biotechnol.* April 2017;35(4):316–319. 10.1038/nbt.3820 28398311

[ref68] Didelot X Parkhill J : A scalable analytical approach from bacterial genomes to epidemiology. *Philos. Trans. R Soc. Lond. B Biol. Sci.* October 2022;377(1861):20210246. 0962-8436, 1471-2970. 10.1098/rstb.2021.0246 35989600 PMC9393561

[ref4] Djaffardjy M Marchment G Sebe C : Developing and reusing bioinformatics data analysis pipelines using scientific workflow systems. *Comput. Struct. Biotechnol. J.* 2023;21:2075–2085. 10.1016/j.csbj.2023.03.003 36968012 PMC10030817

[ref43] Edwards R Katelyn P Daniel S : Linsalrob/PhiSpy: Version 3.4 prerelease. *Zenodo.* October 2019.

[ref13] Ewels PA Peltzer A Fillinger S : The nf-core framework for community-curated bioinformatics pipelines. *Nat. Biotechnol.* February 2020;38(3):276–278. 10.1038/s41587-020-0439-x 32055031

[ref30] Ewels P Magnusson M Lundin S : MultiQC: Summarize analysis results for multiple tools and samples in a single report. *Bioinformatics.* June 2016;32(19):3047–3048. 10.1093/bioinformatics/btw354 27312411 PMC5039924

[ref35] Feldgarden M Brover V Haft DH : Using the NCBI AMRFinder Tool to Determine Antimicrobial Resistance Genotype-Phenotype Correlations Within a Collection of NARMS Isolates. *bioRxiv.* February 2019; page550707. 10.1101/550707

[ref48] Graham ED Heidelberg JF Tully BJ : Potential for primary productivity in a globally-distributed bacterial phototroph. *ISME J.* March 2018;12(7):1861–1866. 10.1038/s41396-018-0091-3 29523891 PMC6018677

[ref3] Grüning B Chilton J Köster J : Practical computational reproducibility in the life sciences. *Cell Systems.* June 2018;6(6):631–635. 10.1016/j.cels.2018.03.014 29953862 PMC6263957

[ref29] Gurevich A Saveliev V Vyahhi N : QUAST: Quality assessment tool for genome assemblies. *Bioinformatics.* April 2013;29(8):1072–1075. 13674803. 10.1093/bioinformatics/btt086 23422339 PMC3624806

[ref26] Haghshenas E Asghari H Stoye J : HASLR: Fast Hybrid Assembly of Long Reads. *iScience.* August 2020;23(8):101389. 10.1016/j.isci.2020.101389 32781410 PMC7419660

[ref65] Holt KE Wertheim H Zadoks RN : Genomic analysis of diversity, population structure, virulence, and antimicrobial resistance in Klebsiella pneumoniae, an urgent threat to public health. *Proc. Natl. Acad. Sci.* July 2015;112(27):E3574–E3581. 0027-8424, 1091-6490. 10.1073/pnas.1501049112 26100894 PMC4500264

[ref71] Janssen L Almeida FM de Damasceno TAS : A Novel Multidrug Resistant, Non-Tn4401 Genetic Element-Bearing, Strain of Klebsiella pneumoniae Isolated From an Urban Lake With Drinking and Recreational Water Reuse. *Front. Microbiol.* November 2021;12:732324. 1664-302X. 10.3389/fmicb.2021.732324 34899623 PMC8654192

[ref36] Jia B Raphenya AR Alcock B : CARD 2017: Expansion and model-centric curation of the comprehensive antibiotic resistance database. *Nucleic Acids Res.* January 2017;45(D1):D566–D573. 13624962. 10.1093/nar/gkw1004 27789705 PMC5210516

[ref60] Jolley KA Maiden MC : BIGSdb: Scalable analysis of bacterial genome variation at the population level. *BMC Bioinformatics.* 2010 December;11(1):595. 1471-2105. 10.1186/1471-2105-11-595 21143983 PMC3004885

[ref21] Koren S Walenz BP Berlin K : Canu: Scalable and accurate long-read assembly via adaptive κ -mer weighting and repeat separation. *Genome Res.* March 2017;27(5):722–736. 15495469. 10.1101/gr.215087.116 28298431 PMC5411767

[ref22] Kolmogorov M Yuan J Yu L Pevzner PA : Assembly of long, error-prone reads using repeat graphs. *Nat. Biotechnol.* 2019;37(5):540–546. 15461696. 10.1038/s41587-019-0072-8 30936562

[ref62] Lahlaoui H De Luca F Maradel S : Occurrence of conjugative IncF-type plasmids harboring the blaCTX-M-15 gene in Enterobacteriaceae isolates from newborns in Tunisia. *Pediatr. Res.* January 2015;77(1):107–110. 0031-3998, 1530-0447. 10.1038/pr.2014.153 25295412

[ref16] Leger A Leonardi T : pycoQC, interactive quality control for Oxford Nanopore Sequencing. *J. Open Source Softw.* 2019;4(34):1236. 10.21105/joss.01236

[ref20] Li D Liu C-M Luo R : MEGAHIT: An ultra-fast single-node solution for large and complex metagenomics assembly via succinct de Bruijn graph. *Bioinformatics.* January 2015;31(10):1674–1676. 10.1093/bioinformatics/btv033 25609793

[ref58] Li W O’Neill KR Haft DH : RefSeq: Expanding the Prokaryotic Genome Annotation Pipeline reach with protein family model curation. *Nucleic Acids Res.* December 2020;49(D1):D1020–D1028. 10.1093/nar/gkaa1105 33270901 PMC7779008

[ref39] Liu B Zheng D Jin Q : VFDB 2019: A comparative pathogenomic platform with an interactive web interface. *Nucleic Acids Res.* November 2018;47(D1):D687–D692. 10.1093/nar/gky1080 30395255 PMC6324032

[ref28] Loman NJ Quick J Simpson JT : A complete bacterial genome assembled de novo using only nanopore sequencing data. *Nat. Methods.* June 2015;12(8):733–735. 10.1038/nmeth.3444 26076426

[ref6] Mölder F Jablonski KP Letcher B : Sustainable data analysis with snakemake. *F1000Res.* April 2021;10:33. 10.12688/f1000research.29032.2 34035898 PMC8114187

[ref70] Murigneux V Roberts LW Forde BM : MicroPIPE: Validating an end-to-end workflow for high-quality complete bacterial genome construction. *BMC Genomics.* June 2021;22(1):474. 10.1186/s12864-021-07767-z 34172000 PMC8235852

[ref8] Olawoye IB Frost SDW Happi CT : The Bacteria Genome Pipeline (BAGEP): An automated, scalable workflow for bacteria genomes with Snakemake. *PeerJ.* 2020;8:e10121. 2167-8359. 10.7717/peerj.10121 33194387 PMC7597632

[ref66] Paczosa MK Mecsas J : Klebsiella pneumoniae: Going on the Offense with a Strong Defense. *Microbiol. Mol. Biol. Rev.* September 2016;80(3):629–661. 10.1128/mmbr.00078-15 27307579 PMC4981674

[ref7] Petit RA Read TD : Bactopia: A Flexible Pipeline for Complete Analysis of Bacterial Genomes. *mSystems.* 2020;5(4). 10.1128/mSystems.00190-20 32753501 PMC7406220

[ref54] Priyam A Woodcroft BJ Rai V : Sequenceserver: A Modern Graphical User Interface for Custom BLAST Databases. *Mol. Biol. Evol.* August 2019;36(12):2922–2924. 10.1093/molbev/msz185 31411700 PMC6878946

[ref46] Puterová J Martínek T . digIS: Towards detecting distant and putative novel insertion sequence elements in prokaryotic genomes. *BMC Bioinformatics.* December 2021;22(1):258. 1471-2105. 10.1186/s12859-021-04177-6 34016050 PMC8147514

[ref9] Quijada NM Rodríguez-Lázaro D Eiros JM : TORMES: An automated pipeline for whole bacterial genome analysis. *Bioinformatics.* April 2019;35(21):4207–4212. 1367-4803. 10.1093/bioinformatics/btz220 30957837

[ref74] Ramírez-Sánchez D Gibelin-Viala C Mayjonade B : Investigating genetic diversity within the most abundant and prevalent non-pathogenic leaf-associated bacteria interacting with Arabidopsis thaliana in natural habitats. *Front. Microbiol.* September 2022;13:984832. 1664-302X. 10.3389/fmicb.2022.984832 36212843 PMC9537739

[ref25] Ruan J Li H : Fast and accurate long-read assembly with wtdbg2. *Nat. Methods.* December 2019;17(2):155–158. 10.1038/s41592-019-0669-3 31819265 PMC7004874

[ref10] Ruiz-Perez CA Conrad RE Konstantinidis KT : MicrobeAnnotator: A user-friendly, comprehensive functional annotation pipeline for microbial genomes. *BMC Bioinformatics.* January 2021;22(1):11. 10.1186/s12859-020-03940-5 33407081 PMC7789693

[ref40] Sayers S Li L Ong E : Victors: A web-based knowledge base of virulence factors in human and animal pathogens. *Nucleic Acids Res.* October 2018;47(D1):D693–D700. 10.1093/nar/gky999 30365026 PMC6324020

[ref69] Schwengers O Hoek A Fritzenwanker M : ASA3P: An automatic and scalable pipeline for the assembly, annotation and higher-level analysis of closely related bacterial isolates. *PLoS Comput. Biol.* March 2020b;16(3):e1007134–e1007115. 10.1371/journal.pcbi.1007134 32134915 PMC7077848

[ref32] Schwengers O Jelonek L Dieckmann MA : Bakta: Rapid and standardized annotation of bacterial genomes via alignment-free sequence identification. *Microb. Genom.* November 2021;7(11). 2057-5858. 10.1099/mgen.0.000685 PMC8743544 34739369

[ref34] Schwengers O Barth P Falgenhauer L : Platon: Identification and characterization of bacterial plasmid contigs in short-read draft assemblies exploiting protein sequence-based replicon distribution scores. *Microb. Genom.* October 2020a;6(10). 10.1099/mgen.0.000398 32579097 PMC7660248

[ref31] Seemann T : Prokka: Rapid prokaryotic genome annotation. *Bioinformatics.* July 2014;30(14):2068–2069. 14602059. 10.1093/bioinformatics/btu153 24642063

[ref57] Simão FA Waterhouse RM Ioannidis P : BUSCO: Assessing genome assembly and annotation completeness with single-copy orthologs. *Bioinformatics.* June 2015;31(19):3210–3212. 10.1093/bioinformatics/btv351 26059717

[ref24] Shafin K Pesout T Lorig-Roach R : Nanopore sequencing and the Shasta toolkit enable efficient de novo assembly of eleven human genomes. *Nat. Biotechnol.* May 2020;38(9):1044–1053. 10.1038/s41587-020-0503-6 32686750 PMC7483855

[ref11] Sserwadda I Mboowa G : rMAP: The Rapid Microbial Analysis Pipeline for ESKAPE bacterial group whole-genome sequence data. *Microbial Genomics.* June 2021;7(6). 2057-5858. 10.1099/mgen.0.000583 PMC8461470 34110280

[ref41] Starikova EV Tikhonova PO Prianichnikov NA : Phigaro: High throughput prophage sequence annotation. *bioRxiv.* April 2019; page598243. 10.1101/598243 32311023

[ref63] Tian D Wang M Zhou Y : Genetic diversity and evolution of the virulence plasmids encoding aerobactin and salmochelin in *Klebsiella pneumoniae.* *Virulence.* December 2021;12(1):1323–1333. 2150-5594, 2150-5608. 10.1080/21505594.2021.1924019 33970792 PMC8115583

[ref23] Vaser R Šikić M : Time- and memory-efficient genome assembly with Raven. *Nat. Comput. Sci.* May 2021;1(5):332–336. 10.1038/s43588-021-00073-4 38217213

[ref27] Walker BJ Abeel T Shea T : Pilon: An integrated tool for comprehensive microbial variant detection and genome assembly improvement. *PLoS One.* November 2014;9(11):e112963. 19326203. 10.1371/journal.pone.0112963 25409509 PMC4237348

[ref14] Wick RR Judd LM Gorrie CL : Completing bacterial genome assemblies with multiplex MinION sequencing. *Microbial. Genomics.* October 2017a;3(10). 10.1099/mgen.0.000132 29177090 PMC5695209

[ref19] Wick RR Judd LM Gorrie CL Holt KE : Unicycler: Resolving bacterial genome assemblies from short and long sequencing reads. *PLoS Comput. Biol.* June 2017b;13(6):e1005595. 15537358. 10.1371/journal.pcbi.1005595 28594827 PMC5481147

[ref5] Wratten L Wilm A Göke J : Reproducible, scalable, and shareable analysis pipelines with bioinformatics workflow managers. *Nat. Methods.* September 2021;18(10):1161–1168. 10.1038/s41592-021-01254-9 34556866

[ref51] Xie Y Dervieux C Riederer E : *R Markdown Cookbook.* Boca Raton, Florida: Chapman and Hall/CRC;2020.

[ref1] Xuan J Ying Y Qing T : Next-generation sequencing in the clinic: Promises and challenges. *Cancer Lett.* November 2013;340(2):284–295. 10.1016/j.canlet.2012.11.025 23174106 PMC5739311

[ref61] Zheng R Zhang Q Guo Y : Outbreak of plasmid-mediated NDM-1-producing Klebsiella pneumoniae ST105 among neonatal patients in Yunnan, China. *Ann. Clin. Microbiol. Antimicrob.* February 2016;15(1):10. 10.1186/s12941-016-0124-6 26896089 PMC4761218

