# Supplementary material for: Scalable and versatile container-based pipelines for de novo genome assembly and bacterial annotation
Source: F1000Res. 2023 Sep 25;12:1205. [Version 1] doi: 10.12688/f1000research.139488.1 (PMC10646344; doi:10.12688/f1000research.139488.1)
Supplement: Peer review history

# Reviewer report: Reviewer response for version 1

**DOI:** 10.5256/f1000research.152764.r219643

**Published:** 2023-11-14

**Authors:** Austin G Davis-Richardson

**Affiliations:** 1 One Codex, San Francisco, California, USA

The authors have developed a suite of open source workflows implemented in Nextflow for microbial WGS preprocessing, de novo assembly, and annotation. The goal of this work is to provide re-usable workflows that can be tailored to the end-user's needs, therefore several interchangeable tools (e.g., de novo assemblers) are wrapped in a common Nextflow framework. Interoperability between bioinformatics tools is a significant challenge therefore these workflows provide a useful resource for tailoring a set of tools to meet one's needs based on sequencing technology.

 The workflows are then applied to a real world problem of analyzing genomic sequences from a Klebsiella isolate with recapitulation of antibiotics resistance phenotypes from genomic data as the "ground truth" for validating the workflow. While this is a limited dataset, the purpose of the paper is to not validate all of the bioinformatics tools used in the workflow but to demonstrate how it could be used to generate a useful output from genomic sequencing data.

 The paper would benefit from either demonstrating the first two pipelines (excluding annotation) on a non-bacterial dataset as the authors claim that only the annotation pipeline is prokaryote-specific.

 I was able to download and start the Nextflow pipelines however I do not have access to sufficient computational resources required to complete the pipelines. Therefore, I am unable to evaluate the outputs of the pipelines at this time. It would be useful for the authors to provide example outputs on Zenodo.

Are the conclusions about the tool and its performance adequately supported by the findings presented in the article?

Yes

Is the rationale for developing the new software tool clearly explained?

Yes

Is the description of the software tool technically sound?

Yes

Are sufficient details of the code, methods and analysis (if applicable) provided to allow replication of the software development and its use by others?

Yes

Is sufficient information provided to allow interpretation of the expected output datasets and any results generated using the tool?

Partly

Reviewer Expertise:

Metagenomics, Microbial Genomics, Microbiome

I confirm that I have read this submission and believe that I have an appropriate level of expertise to confirm that it is of an acceptable scientific standard.

# Reviewer report: Reviewer response for version 1

**DOI:** 10.5256/f1000research.152764.r218196

**Published:** 2023-11-14

**Authors:** Abhinav Sharma, Emilyn Costa Conceição

**Affiliations:** 1 Department of Biomedical Sciences, Stellenbosch University, Stellenbosch, Western Cape, South Africa

**Summary:**

 The authors present 3 modular piplines aimed at (non-)bioinformaticians and applicable in different stages of bacterial analysis such as data download, assembly and annotation and generates reports to facilitate the interpretation of the results. The pipelines also support the analysis of data from multiple sequencing platforms, aiding the reuse of the pipeline for both short and long read sequences, which adds value to the bioinformatical toolkit given the increasing use of long-read sequences and the current prevalence of short-read sequences.

 Furthermore, these pipelines have been written in Nextflow and generally follow the best practices of modularization outlined by the nf-core community pipeline template. 

**Suggestion (ref documentation website):**

 As far as the design of the pipeline and description of the design choices goes, it is well explained. The interpretation of the generated results has been neatly described for bacannot in the documentation page (https://bacannot.readthedocs.io/en/latest/outputs/), including the directory structure and the relevant links for the tool-specific reference material. However as the pipelines are modular and the user is expected to run them separately, it is recommended that the documentaiton websites for MpGAP pipeline and ngs-preprocess pipelines should also have an "Output" page to facilitate users on the output structure and refer the correct tools-specific links.

Are the conclusions about the tool and its performance adequately supported by the findings presented in the article?

Yes

Is the rationale for developing the new software tool clearly explained?

Yes

Is the description of the software tool technically sound?

Yes

Are sufficient details of the code, methods and analysis (if applicable) provided to allow replication of the software development and its use by others?

Yes

Is sufficient information provided to allow interpretation of the expected output datasets and any results generated using the tool?

Partly

Reviewer Expertise:

Mycobacterium tuberculosis, Reproducible methods, Machine learning, Variant calling, Reference Alignment, Resistance profiling and Scientific workflow managers.

We confirm that we have read this submission and believe that we have an appropriate level of expertise to confirm that it is of an acceptable scientific standard.

# Reviewer report: Reviewer response for version 1

**DOI:** 10.5256/f1000research.152764.r210163

**Published:** 2023-10-18

**Authors:** Abdolrahman Khezri

**Affiliations:** 1 Innland Norway university of applied sciences, Hamar, Norway

The paper entitled "Scalable and versatile container-based pipelines for de novo genome assembly and bacterial annotation", takes advantage of several well-known tools for NGS data processing, assembly, and downstream analyses. The GitHub page seems to provide enough information too. However, the paper still needs to improve in some aspects. 

 The aim of this paper is not clear. The authors developed a pipeline based on existing tools. However, the aim is not easy to find, and the work lacks novelty. 

 The other drawback of this paper is the fact that the authors tested the pipeline only using a clinical isolate. How this pipeline handles, for instance, gram-positive bacteria or other bacteria with different genome sizes is not clear. 

 The figures are not accurate. They do not provide enough/precise information regarding the steps, tasks, or tools. 

 It is necessary to implement the Bandage tool into the pipeline right after assembly to visualize the assembly.

 Is there any specific reason the author chose fastp as a quality check tool? Obviously, fastqc provides much more details compared to fastp.

 I understand that different tools make different assembly, however many publications already benchmarked them, for instance, it is well known that SPAdes, Flye, and Unicycler provides a better assembly for short, long, and hybrid assembly, respectively. Please see Wick *et al. *(2019 ^1^) or Khezri *et al. *(2021 ^2^). However, in my opinion in a pipeline, an author should stick to the best overall tool and avoid providing many tools as pipelines are designed for less experienced users.

 In Table 4, some of the tasks such as annotation with Prokka as well as Quast and Busco took a tremendous amount of time which is not how it should be. What is the author's comment on this?

 Although the authors presented the differences between their pipeline and other published pipelines, the samples in this study should be tested using other pipelines in order to have a clear demonstration of performance. 

 Porechop is out of date and no longer supported. This is important when it comes to new chemistry and sequencing kits, where, for instance, 24 barcodes in rapid barcoding kits are not recognized by Porechop. Here it would be better to take advantage of ONT guppy software not only for trimming but also for other tasks. 

 Very little is written regarding BLAST. How can the user benefit from BLAST in this pipeline? Does it come with ore-indexed reference databases?

 The Prokka output can be used for many different purposes including core genome and pan genome analyses which are important for people working on epidemiology. It would be nice if the author could extend their pipeline to cover this.

Are the conclusions about the tool and its performance adequately supported by the findings presented in the article?

No

Is the rationale for developing the new software tool clearly explained?

Partly

Is the description of the software tool technically sound?

Yes

Are sufficient details of the code, methods and analysis (if applicable) provided to allow replication of the software development and its use by others?

Partly

Is sufficient information provided to allow interpretation of the expected output datasets and any results generated using the tool?

Partly

Reviewer Expertise:

Bioinformatics and bacterial genomics

I confirm that I have read this submission and believe that I have an appropriate level of expertise to confirm that it is of an acceptable scientific standard, however I have significant reservations, as outlined above.

## References

[1] : Benchmarking of long-read assemblers for prokaryote whole genome sequencing. *F1000Res* .2019;8: 10.12688/f1000research.21782.4 2138 10.12688/f1000research.21782.4 31984131PMC6966772

[2] : Hybrid Assembly Provides Improved Resolution of Plasmids, Antimicrobial Resistance Genes, and Virulence Factors in Escherichia coli and Klebsiella pneumoniae Clinical Isolates. *Microorganisms* .2021;9(12) : 10.3390/microorganisms9122560 10.3390/microorganisms9122560 34946161PMC8704702
